# Effects of saliva contamination and decontamination procedures on shear bond strength of self-etch dentine bonding systems: An *in vitro* study

**DOI:** 10.4103/0972-0707.66714

**Published:** 2010

**Authors:** Krishna Neelagiri, M Kundabala, Rashmi A Shashi, Manuel S Thomas, Abhishek Parolia

**Affiliations:** Department of Conservative Dentistry and Endodontics, Manipal College of Dental Sciences, Manipal, India; 1 Department of Conservative Dentistry and Endodontics, Manipal College of Dental Sciences, Mangalore, Karnataka, India

**Keywords:** Decontamination procedure, salivary contamination, self-etch adhesive system, shear bond strength

## Abstract

**Objective:**

This study aims to evaluate the effect of saliva contamination on the shear bond strength of two self-etch dentine bonding systems and also investigate the effect of decontamination procedure on the recovery of bond strength.

**Materials and Methods:**

Sixty premolars extracted for orthodontic reason were obtained and the buccal surfaces of teeth were reduced to create a flat dentine surface. The samples were randomly divided into three sub-groups for AdheSE (ASE) (Ivoclar – Vivadent, Schaan, Liechtenstein) and three sub-groups for Adper Prompt Self-Etch Adhesive (ADP) (3M ESPE, St Paul, MN, USA) of 10 each. For AdheSE (ASE); ASE-I was the control group (primer applied to fresh dentine surface), ASE-II was the contamination group (primer applied, followed by saliva contamination and then air dried) and ASE-III was the decontamination group (primer applied, followed by saliva contamination, air dried and then primer reapplied). For Adper Prompt (ADP); ADP-I was the control group (self-etch adhesive applied to fresh dentine surface), ADP-II was the contamination group (self-etch adhesive applied, followed by saliva contamination and then air dried) and ADP-III was the decontamination group (self-etch adhesive applied, followed by saliva contamination, air dried and then self-etch adhesive reapplied). Followed by the bonding procedure, a 5 mm composite resin block with Filtek P-60 (3M ESPE, St Paul, MN, USA) was built on the substrate. Shear bond strength (SBS) was tested with Instron Universal testing machine (Instron Corporation, Canton, MA, USA) with a cross head speed of 1 mm per minute. Data obtained was subjected to one way ANOVA test, while the inter group comparison was made using Tukey’s multiple comparison and Unpaired *t*-test.

**Results:**

In AdhSE group (ASE), the sub-group ASE-II (contamination group) [5.4 ± 2.2 MPa] showed lower SBS than ASE-I [11.8 ± 2.6 MPa] and ASE-III [8.9 ± 3.3 MPa], which was statistically significant. There was no significant difference in the bond strength between the ASE-I (control group) and ASE-III (decontamination group). In Adper Prompt group (ADP), there was a severe decrease of bond strength in ADP-II (contamination group) [4.6 ± 1.1 MPa] when compared to ADP-I (control group) [7.4 ± 1.4 MPa] and ADP-III (decontamination subgroup) [14.1 ± 2.2 MPa] which was statistically significant. The bond strength of ADP-III wherein Adper Prompt bonding agent was reapplied after salivary contamination was found to be statistically significant than ADP-I and ADP-II.

**Conclusion:**

Saliva contamination reduces the dentine bond strength of both the self-etch systems; AdheSE and Adper Prompt. Re-application of the primer for the AdheSE and re-application of the adhesive for the Adper Prompt after air drying the saliva off can recover the dentine bond strength. In the Adper Prompt group, the added application of adhesives to decontaminate saliva not only recovered the bond strength but also improved it significantly.

## INTRODUCTION

Adhesion to dentine has been a subject of considerable interest because it is a more heterogeneous substrate with much higher organic and water content than enamel.[1] The condition of the tooth structure and the chemical composition of the adhesive agent have shown to affect the bond strength.[2] Hence, improving adhesive restorative materials has been the objective of research in the recent years.

Clinically, there are many factors that affect adhesion and retention of resin-containing restorative materials. Moisture such as gingival fluid, blood, hand-piece oil[3] and, in particular saliva, can affect the quality of the bond, leading to micro-leakage at the tooth restoration interface. This may result in the loss of restoration, recurrent caries, postoperative sensitivity and discoloration.[4] Therefore the bonding procedure requires proper isolation and prevention of contamination. However, many carious lesions which require the use of dentine bonding agents are found in the areas that are difficult to isolate, especially when the site is near or at the gingival margin where saliva contamination is more likely to occur.[5]

Silverstone *et al*.[6] have reported that saliva contamination of etched enamel caused a significant decrease in bond strength between the resin and enamel surface. It was suggested that the contamination of etched enamel by salivary proteins prevented monomers from penetrating the pores in enamel, which reduced the bond strength.[3] Microscopic examination of saliva contaminated acid-etched enamel showed the formation of an organic pellicle that could not be removed with water.[6] The organic pellicle coating masked the underlying enamel pores, decreased resin accessibility and impaired mechanical adhesion. However, the contaminated enamel could be reconditioned by an additional 10 seconds of acid etching.[7]

Dentine adhesion is extremely complex when compared to enamel bonding. The micromechanical resin adhesion to dentine differs fundamentally from the relatively simple interlocking of bonding agents with enamel. Therefore the result of many studies related to the bonding efficacy of saliva contaminated dentine bonding agents has varied.[1] Fritz, Finger and Stean[8] found that saliva contamination of cured one-bottle type adhesive resulted in low shear bond strengths and wide marginal gaps. On the contrary, others reported that the saliva contamination of dentine had no adverse effect on the bonding efficiency of one-bottle adhesive systems.[9–11]

Recently developed adhesive systems such as the “self-etching primers” and the ”self-etch adhesives” have shown to be resistant to salivary contamination.[4,12] Self-etch systems eliminate the rinsing and drying steps which simplifies the bonding procedure. In addition to this, the possibility of over wetting or over drying is reduced, which in turn helps in better adhesion.[13–15] These dentine bonding agents have a reduced number of components and application steps and this reduces the risk of saliva contamination in the field of operation.[16]

Hence this study was conducted to evaluate the influence of saliva contamination of dentine during the bonding procedure on shear bond strength and to investigate the effect of contamination removing treatment on the recovery of bond strength of two self-etch dentine bonding systems.

## MATERIALS AND METHODS

We tested two dentine adhesives in the study: AdheSE (Ivoclar – Vivadent, Schaan, Liechtenstein) and Adper Prompt Self-Etch Adhesive (3M ESPE, St Paul, MN, USA) [Table 1]. Filtek P-60 posterior restorative composite resin (3M ESPE, St Paul, MN, USA) was used in both the groups. Sixty premolars extracted for orthodontic reasons were obtained for the shear bond test. The teeth were debrided, cleaned and stored in isotonic saline, until use. The teeth were sectioned at the cementodentinal junction and the coronal portion of the teeth was embedded in acrylic with the buccal surface facing outwards. The buccal surface of the teeth was reduced to create flat dentine surface with a medium grit diamond bur using high-speed handpiece under air water spray. The specimens were then randomly divided into two groups (AdheSE (ASE) group and Adper Prompt (ADP) group) of 30 samples each.

**Table 1:** Chemical composition of the bonding systems tested

Product name	Manufacturer	Composition	Application
AdheSE	Ivoclar – Vivadent Schaan, Liechtenstein	Primer: dimethacrylate, phosphoric acid acrylate, initiators, stabilizers, Water bond: HEMA, dimethacrylate, silicon dioxide, initiators	Apply self-etch primer for at least 30 seconds. Remove excess of primer with air. Apply the bonding agent and gently air blow. Light cure for 10 seconds.
Adper Prompt	3M ESPE St Paul, MN USA	Liquid 1: methacrylated phosphoric esters, BisGMA, camphorquinone, stabilizers Liquid 2: water, HEMA, polyalkenoic acid, stabilizers	Mix two solutions (A and B) for five seconds, apply the mixture for 15 seconds, gently air dry and light cure for 10 seconds.

For each adhesive, the specimens were divided into non-contaminated (which was the control group), contaminated and decontaminated sub-groups (which were the experimental groups). Ten specimens were made for each procedure. In the experimental groups, fresh whole saliva was applied to the surface of the specimens with a disposable brush for 5 seconds, followed by the contaminant removing treatment, if applicable. Details of the bonding procedure for each adhesive are presented. Following the bonding procedure, a 5 mm composite resin block was built on the substrate using a plastic tube having an internal diameter of 4.9 mm by progressively adding 1.5 to 2 mm thick increments. In order to ensure the proper polymerization of each added layer of composite resin, the light tip was positioned as close as possible to the surface.

After polymerization, the teeth were sectioned 1 mm apical to cemento-enamel junction and the specimens were mounted in cylindrical molds with self-curing acrylic resins. Shear bond strength was tested with an Instron Universal testing machine (Instron Corporation, Canton, MA, USA). Each specimen was mounted in a shear testing apparatus and a chisel shaped shearing rod with a cross-head speed of 1 mm per minute was used to load the specimens at the dentine-composite interface. The shear bond strength data was subjected to One-way ANOVA test, while the intergroup comparison was made using Tukey multiple comparison and Unpaired *t*-test.

## RESULTS

**Figure 1:**
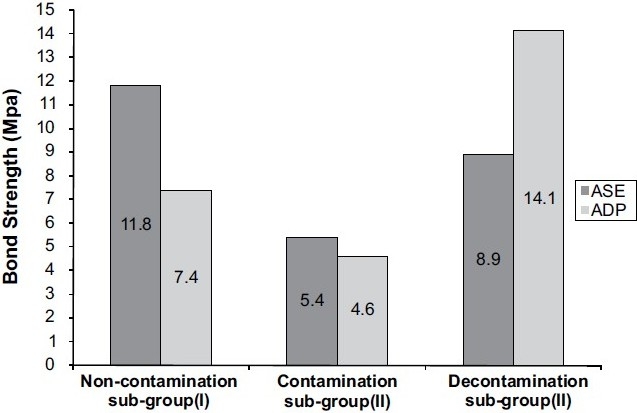
Inter-group comparison of ASE and ADP

The results of the shear bond strength test to AdheSE (ASE) and Adper Prompt (ADP) are shown in Tables 2 and 3 and Figure 1. One-way ANOVA test revealed a significant difference at *P* <0.05. Comparisons within the groups ASE and ADP are shown in Tables 4 and 5.

**Table 2:** Mean bond strength values (MPa) of AdheSE group

Group	Sub-group	N	Mean SBS ± SD
ASE	Non-contaminated group (ASE-I)	10	11.8 ± 2.6
	Contaminated group (ASE-II)	10	5.4 ± 2.2
	Decontaminated group (ASE-III)	10	8.9 ± 3.3

**Table 3:** Mean bond strength values (MPa) of Adper Prompt

Group	Sub-group	N	Mean ± SD
ADP	Non-contaminated group (ADP-I)	10	7.4 ± 1.5
	Contaminated group (ADP-II)	10	4.6± 1.1
	Decontaminated group (ADP-III)	10	14.1 ± 2.2

**Table 4:** Comparisons among the AdheSE group (ASE).

Group	(A)Subgroup	(B) Subgroup	Mean difference (A-B)	*P* value
ASE	Non-contaminated group (ASE-I)	Contaminated group (ASE-II)	6.4	0.001 vhs***
		Decontaminated group (ASE-III)	2.9	0.064 ns*
	Contaminated group (ASE-II)	Decontaminated group (ASE-III)	-3.5	0.019 sig**

* ns- not significant,

** sig- significant,

*** vhs- very highly significant

**Table 5:** Comparisons among the Adper Prompt group

Group	(A)Subgroup	(B) Subgroup	Mean difference (A-B)	*P* value
ADP	Noncontaminated group (ADP-I)	Contaminated group (ADP-II)	2.7	0.003 vhs***
		Decontaminated group (ADP-III)	-6.7	0.001 vhs***
	Contaminated group (ASE-II)	Decontaminated group (ADP-III)	-9.5	0.001 vhs***

* ns- not significant,

** sig- significant,

*** vhs- very highly significant

In the AdheSE group (ASE), the difference was very highly significant (*P* = 0.001) between the control (ASE-I) and the contamination sub-group (ASE-II). The shear bond strength decreased to 5.4 ± 2.2 MPa when compared to 11.8 ± 2.6 MPa of control group. However, when the self -etch primer was reapplied after the salivary contamination (ASE-III), the bond strength increased to 8.9 ± 3.3 MPa (*P* = 0.019). There was no significant difference in the bond strength between the control and the decontamination group (*P* = 0.064).

In Adper Prompt group (ADP), when the dentine was not contaminated (ADP-I), the shear bond strength was 7.4 ± 1.5 MPa. When the salivary contamination occurred after the curing of the bonding agent (ADP-II), the bond strength decreased to 4.6 ± 1.1 MPa. This decrease in bond strength was highly significant (*P*= 0.003). The bond strength of ADP-III where Adper Prompt bonding agent was reapplied after salivary contamination was 14.1 ± 2.2 MPa. This was statistically very highly significant than the control group and the group where salivary contamination was done (*P*= 0.001).

Intergroup comparisons between ASE and ADP were done using Unpaired *t*-test. Table 6 shows the comparison of bond strength when the specimens were not contaminated with saliva. There was statistically very highly significant difference between the mean bond strengths of ASE-I and ADP-I (*P*=0.001). The control specimens of AdheSE group showed higher bond strength than that of Adper Prompt group.

**Table 6:** Comparisons of mean bond strengths between ASE-I (control) and ADP-I (control)

Subgroup	Group	N	Mean ± SD	*P* value
Control	ASE-I	10	11.8 ± 2.6	0.001 vhs***
	ADP-I	10	7.4 ± 1.5	

* ns- not significant,

** sig- significant,

*** vhs- very highly significant

## DISCUSSION

The laboratory parameter most often measured in dentine adhesion is shear bond strength. Flat dentine surfaces are prepared in the extracted tooth, the adhesive system is applied and the composite resin cylinder is bonded over the adhesive. A shear force is then applied at the resin–dentine interface, using a knife-edge probe. The shear bond strength test is only a rough tool for evaluating the relative efficacy of bonding materials. Never the less, they are excellent for screening new materials and for comparing the same parameter among different adhesive systems.[17]

Saliva contamination of operating field is a frequent problem in restorative procedures, especially when rubber dam isolation is difficult or impossible, such as in the case of deep cervical lesions or to seat an indirect restoration or even in patients having problem in opening their mouth. In the present study natural saliva was chosen as the contaminant because artificial saliva may confound the results. In addition, many studies have accepted whole healthy human saliva as an acceptable contaminating medium. An *in vitro* model to mimic clinical conditions proved that saliva and plasma to be detrimental to hybrid layer formation.[8]

Effect of saliva contamination is a matter of great controversy. Few studies have reported that the use of dentine bonding agents under fissure sealants has reduced their sensitivity to saliva contamination and provided high bond strengths.[18,19] Some have reported that the saliva contamination of dentine had no adverse effect on the bonding efficiency of one-bottle adhesive systems.[9–11] Others have shown that the saliva contamination of the dentine surface produced a significant decrease in the bond strength.[3,8,17,20]

The factors that can be hypothesized as the cause for reduction in the bond strength in saliva contaminated dentine are as follows:[8,21]

1. Adsorption of glycoprotein to the poorly polymerized adhesive surface where they might act as a barrier that prevents complete wetting with the next increment of resin and thus prevent adequate co-polymerization.
2. Salivary proteins might prevent monomers from penetrating the collagen network of dentine or there can be an increase in the contact angle which could decrease the bond strength.
3. Excess saliva may dilute the primer and thus produce a weak hybrid layer.
4. Co-polymerization with the subsequent resin layer could be compromised by the removal of the oxygen inhibited unpolymerized surface layer. This hypothesis is not likely as investigators have shown that there is no difference in bond strength when resin composite is added and polymerized on cured adhesive with or without an unpolymerized surface layer.

Ghavam and Pour[20] showed that there was no significant difference when the contaminated dentine was either washed or washed and re-etched. Fritz *et al*[8] showed that re-etching is not necessary when contamination with the saliva happens. EL -Kalla and Godoy[12] believed that when saliva contamination happens after etching the dentine, blot drying the surface achieves bond strength equal to that of the uncontaminated group. Studies have also shown that the reapplication of primer/ adhesive to improve the bonding efficiency after saliva contamination.[22,23]

The hydrophilic nature of the newer dentine bonding agents may allow them to function to some degree in the presence of saliva contamination by displacing or diffusing through it and then they infiltrate and polymerize within the exposed collagen bundles of demineralized superficial dentine. AdheSE is a relatively new self- etching system containing a primer composed of phosphonic acid acrylate, bis-acrylamide, water, initiators and stabilizers. It also contains a bonding component composed of dimethacrylate, HEMA and highly dispersed silicon-dioxide, initiators and stabilizers. In the AdheSE group, when the saliva contamination was done after the application of self etching primer, bond strength decreased significantly. However, the bond strength could be recovered after reapplication of the primer. This result was in agreement with the study done by Park and Lee.[17]

Adper Prompt Self-Etch Adhesive is based on the original Prompt-L-Pop adhesive and is composed of methacrylated phosphonic esters, BisGMA, initiators, stabilizers, water, HEMA and poly alkenoic acid. In Adper Prompt group, there was a significant decrease in bond strength when saliva contamination occurred after curing the bonding agent. This finding confirms the observation of Fritz and others[8] who have shown that saliva contamination of the cured adhesive layer of one-bottle adhesive system has a detrimental effect on bond strength.

Studies have shown that saliva contamination reduces the dentine bond strengths of all-in-one adhesives and supplementary application of the adhesive after cleaning the saliva from the dentine surface is an empirical recommendation for restoring bond strength.[22,23] In contrast to the results of this study, Ghavan and Pour[20] have shown that contamination of a single bottle system after curing of the adhesive did not decrease the bond strength compared to the control group.

An interesting finding in Adper Prompt group was that the bond strength obtained in the sub-group where the bonding agent was reapplied after saliva contamination was higher than the control group. This increase in bond strength was very highly significant (*P*= 0.001). The increased bond strength could be due to the effect of multiple adhesive coatings. The increased resin-dentine bond strength under multiple applications could be due to several mechanisms operating simultaneously. As the solvent is evaporated the concentration of the co-monomers that exists after each coating increases. This improves the hybrid layer and the ratio of the polymerized vs. unpolymerized adhesive layer due to oxygen inhibition. Hashimoto and others[24–26] have hypothesized that when multiple coats of hydrophilic adhesive solutions are applied, it can displace or diffuse through the film to reach the underlying layer and improve the bond strength.

Intergroup comparison of ASE-I (control) and ADP-I (control) showed that AdheSE has higher bond strength to that of Adper Prompt. This difference in the bond strength was very highly significant (*P*= 0.001). The low bond strength obtained with this system may be due to an incomplete infiltration of the acidic monomers and subsequent partial dissolution of the smear layer, suggesting inconsistent performance in terms of achieving a quality bond.[27]

Further studies have to be conducted to prove these results.

## CONCLUSIONS

The following conclusions can be drawn within the limitations of this *in vitro* study;

1. The self etch primer [AdhSE (ASE-I)] showed better bond strength than self-etch adhesive [Adper Prompt (ADP-I)] when not contaminated with saliva.
2. Salivary contamination reduced the dentine bond strength of both the self-etch primer (ASE-II) and adhesives (ADP-II).
3. Re-application of the primer for the AdhSE group (ASE-III) and re-application of the adhesive for the Adper Prompt group (ADP-III), after air drying the saliva off, can recover the dentine bond strength.
4. In the Adper Prompt group, the added application of adhesives to decontaminate (ADP-III), not only recovered the bond strength but also improved it significantly, which could be attributed to the effect of multiple adhesive coatings
